# Author Correction: Long-duration electricity storage needs for coping with Dunkelflaute events in Europe

**DOI:** 10.1038/s41467-026-75342-9

**Published:** 2026-07-09

**Authors:** Martin Kittel, Alexander Roth, Wolf-Peter Schill

**Affiliations:** 1 German Institute for Economic Research (DIW Berlin), Department of Energy, Transportation, Environment, Anton-Wilhelm-Amo-Strasse 58, 10117 Berlin, Germany; 2 Technical University Berlin, Digital Transformation in Energy Systems, Einsteinufer 25 (TA 8), 10587 Berlin, Germany; 3 Bruegel, Rue de la Charité 33, 1210 Saint-Josse-ten-Noode, Belgium

**Keywords:** Energy supply and demand, Energy economics, Energy security

Correction to: *Nature Communications* 10.1038/s41467-026-72681-5, published online 9 May 2026

Following publication, we identified minor inconsistencies between the published manuscript and the final model used for the publication. In early-stage models that we used before submission, we assumed that hydrogen that is endogenously produced within the modeled European region would not only be used in the power sector via reconversion to serve electricity demand but also to serve an assumed future hydrogen demand in industry, transport and other sectors, based on TYNDP 2022 levels. In the analysis, we decided to restrict hydrogen explicitly to power sector use and abstracted from potential future hydrogen needs of other sectors. To correct for remnants of the earlier parameterization, the following changes have been made.

For Fig. 2, with future hydrogen demand removed, the long-term duration storage capacity has been lowered, as shown in the revised plots, while in the figure caption “and hydrogen” has been removed from the original text “…annual demand for electricity (including electrified heating) and hydrogen.” In the Results, the sentence “Long-duration storage further remains necessary to continuously meet the flat hydrogen demand from coupled sectors, as assumed in our model” has been removed. In the Methods, “and hydrogen” has been removed from the original sentence “Exogenous model inputs entail techno-economic parameters, such as investment and variable costs, availability time series of wind and solar PV, as well as price-inelastic demand time series for electricity and hydrogen.” In the Methods, the sentence “The profiles are scaled to the annual demand levels of the TYNDP (scenario Distributed Energy for 2050) and are reduced by the electrical energy amount required for the domestic generation of the hydrogen that is reconverted to electricity” now replaces the original “…and are reduced by the electrical energy amount required for the generation of the exogenous hydrogen demand.” The Fig. 11 schematic has been amended to now include “oil with CCS” in the energy system. The Supplementary Information has also been updated with associated changes.

For comparison, the original Fig. 2 is available as Supplementary Information accompanying this amendment. The figures, text and Supplementary Information have been updated in the HTML and PDF versions of the article.

## Supplementary information

Original Fig. 2

