# Supplementary material for: Long-duration electricity storage needs for coping with Dunkelflaute events in Europe
Source: Nat Commun. 2026 May 9;17:4210. doi: 10.1038/s41467-026-72681-5 (PMC13157499; doi:10.1038/s41467-026-72681-5)
Supplement: Supplementary file 1 — Supplementary Information [file 41467_2026_72681_MOESM1_ESM.pdf]

Supplementary Information

Long-duration electricity storage needs for coping with Dunkelflaute events in Europe

Martin Kittel*, Alexander Roth, and Wolf-Peter Schill

*Corresponding author: mkittel@diw.de

Contents

Supplementary Methods . . . 3
Formal definition of the hydrogen module . . . 3
Supplementary Notes . . . 7
Supplementary Note 1 . . . 7
Additional renewable drought illustrations: . . . 7
Supplementary Note 2 . . . 9
Additional insights on the correlation between renewable droughts and long-duration storage energy: . . . 9
Supplementary Note 3 . . . 13
Additional illustrations of the impact of interconnection on long-duration storage energy: . . 13
Supplementary Note 4 . . . 20
Additional information on the impact of firm zero-emission generation: . . . 20
Supplementary Note 5 . . . 25
Additional information on a sensitivity with varying values of lost load: . . . 25
Supplementary Tables . . . 27
Additional information on the cost sensitivity analysis . . . 27

Supplementary Methods

Formal definition of the hydrogen module

In the following, we present the model equations implementing the generation, storage, and transport of renewable hydrogen technologies as well as its re-conversion to electricity. For simplicity, we use only a single technology for each of these features.

Endogenous model variables are given in capital letters and exogenous parameters in lowercase. We denote the electricity demand for the production of green hydrogen production $G^{ely}_{n,ely,t}$ in time step t and country n. Each electrolysis technology ely has a specific efficiency $eta^{ely}_{n,ely} < 1$ and a flat availability $avail^{ely}_{n,ely}$. The electricity demand of an electrolyzer must not exceed its generation capacity $N^{ely}_{n,ely}$:

$$G^{ely}_{n,ely,t} \leq avail^{ely}_{n,ely} * N^{ely}_{n,ely} \tag{1}$$

The produced hydrogen is compressed to a system-wide pressure level, constrained by the compressor capacity $N^{comp,ely}_{n,ely}$ with an efficiency $\eta^{comp,ely}_{n,ely} < 1$, and fed into a hydrogen grid that links generation, storage, and re-conversion units as well as import and export pipelines:

$$eta^{ely}_{n,ely} * G^{ely}_{n,ely,t} \leq avail^{ely}_{n,ely} * N^{comp,ely}_{n,ely} \tag{2}$$

Using a storage technology sto, hydrogen can be stored. Hydrogen that is added to storage $STO^{in}_{n,sto,t}$ has to be compressed. Compression losses are reflected in the charging efficiency $eta^{comp,in}_{n,sto} < 1$. A storage energy balance links the storage state-of-charge $STO^{L}_{n,sto}$ inter-temporally. While there might be self-discharge $eta^{sto,self}_{n,sto} < 1$, we assume storage discharge $STO^{out}_{n,sto,t}$ to be lossless due to the high-pressure level:

$$STO^{L}_{n,sto,t} = eta^{sto,self}_{n,sto} * STO^{L}_{n,sto,t-1} + eta^{comp,in}_{n,sto} * STO^{in}_{n,sto,t} - STO^{out}_{n,sto,t} \tag{3}$$

To avoid free lunch, we require that the storage levels are equal in the first time step $t = 1$ and last time step $t = T$:

$$STO^{L}_{n,sto,1} = eta^{sto,self}_{n,sto} * STO^{L,initial,last}_{n,sto} + eta^{comp,in}_{n,sto} * STO^{in}_{n,sto,1} - STO^{out}_{n,sto,1} \tag{4}$$

$$STO^{L}_{n,sto,T} = STO^{L,initial,last}_{n,sto} \tag{5}$$

Suppose $avail^{sto}_{n,sto}$ is the flat availability of each storage technology sto, the storage level cannot exceed the installed storage energy capacity $N^{sto}_{n,sto}$:

$$STO^{L}_{n,sto,t} \leq avail^{sto}_{n,sto} * N^{sto}_{n,sto} \tag{6}$$

To maintain a minimum pressure level required for the cushion gas, we impose a minimum filling state $\phi^{sto,min}_{n,sto} < 1$:

$$STO^{L}_{n,sto,t} \geq \phi^{sto,min}_{n,sto} * avail^{sto}_{n,sto} * N^{sto}_{n,sto} \tag{7}$$

Note that in this paper, we abstract from minimum filling levels to focus on required the working gas, i.e., $\phi^{sto,min}_{n,sto} = 0$. Hourly storage charging is constrained by the compression capacity $N^{comp,sto}_{n,sto}$:

$$STO^{in}_{n,sto,t} \leq avail^{sto}_{n,sto} * N^{comp,sto}_{n,sto} \tag{8}$$

Hourly storage discharge is constrained by a maximum discharge rate $\phi^{sto,max}_{n,sto} < 1$:

$$STO^{out}_{n,sto,t} \leq \phi^{sto,max}_{n,sto} * avail^{sto}_{n,sto} * N^{sto}_{n,sto} \tag{9}$$

Hydrogen can be re-converted to electricity. Suppose $avail^{recon}_{n,recon}$ is the flat availability of the re-conversion technology $recon$ and $\eta^{recon}_{n,recon} < 1$ the conversion efficiency, the electricity output $G^{recon}_{n,recon,t}$ is then limited by capacity of the re-conversion unit $N^{recon}_{n,recon}$:

$$G^{recon}_{n,recon,t} \leq avail^{recon}_{n,recon} * N^{recon}_{n,recon} \tag{10}$$

Hydrogen can be generated domestically, imported from other world regions, or exchanged across modeled countries based on a simple cross-border transport model. Hydrogen flows are denoted $F_{p,t}$. The set p consists of all pipelines within the modeled area and those import pathways $path$ from outside the modeled area. The latter include pipeline-based imports from North Africa or Ukraine and ship-based imports from the world market. A hydrogen flow may not exceed its pipeline or shipping capacity N^{trans}_{p}:

$$F_{p,t} \leq N^{trans}_{p} \tag{11}$$

We assume constant hydrogen imports IM_{path}^{const} from outside the modeled area:

$$F_{path,t} = IM_{path}^{const} \tag{12}$$

The sum of all imports accumulates to IM_{path}:

$$\sum_{t} F_{path,t} = IM_{path} \tag{13}$$

To avoid infeasibility, we allow for slack hydrogen generation $INFES_{n,t}$ which needs to equal $INFES_{n}^{const}$ in all time steps to emulate constant imports in settings with disabled hydrogen flows:

$$INFES_{n,t} = INFES_{n}^{const} \tag{14}$$

Lines and countries are linked via the grid and import pathways, which are represented by the directed incidence matrices $inc_{p,n}^{import}$ and $inc_{p,n}^{export}$. Exports incur losses, represented by the transport efficiency $\eta_{p}^{trans} < 1$. The model setup allows to specify an exogenous hydrogen demand $d_{n,t}^{h2}$ for unspecified applications in the industry, transport, or heating sectors. In this model application, however, we set $d_{n,t}^{h2} = 0$ to focus on hydrogen storage needs for long-duration electricity storage. The hydrogen balance of each country n ensures that the hydrogen supply meets hydrogen demand in each time step:

$$\begin{aligned} d_{n,t}^{h2} + \sum_{sto} STO_{n,sto,t}^{in} + \sum_{recon} \frac{G_{n,recon,t}^{recon}}{\eta_{n,recon}^{recon}} + \sum_{p} inc_{p,n}^{export} * \frac{F_{p,t}}{\eta_{p}^{trans}} \\ = \sum_{ely} \eta_{n,ely}^{ely} * \eta_{n,ely}^{comp,ely} * G_{n,ely,t}^{ely} + \sum_{sto} STO_{n,sto,t}^{out} + \sum_{p} inc_{p,n}^{import} * F_{p,t} + INFES_{n,t}. \end{aligned} \tag{15}$$

The hydrogen grid is linked to the electricity grid through the electricity demand for electrolysis $G_{n,ely,t}^{ely}$, hydrogen compression after electrolysis $d_{n,ely}^{comp,ely}$ for injecting into the hydrogen grid, hydrogen compression for injection into storage $d_{n,sto}^{comp,sto}$, and hydrogen booster compression for exporting pipelines $d_{n,p}^{comp,trans}$. For this, we add Supplementary Equation (16) to the demand and Supplementary Equation (17) to the supply side of the electricity balance of each country n, respectively. The latter is documented in Zerrahn and Schill:[1]

$$\ldots+\sum_{ely}(1+\eta^{ely}_{n,ely}*d^{comp,ely}_{n,ely})*G^{ely}_{n,ely,t}+\sum_{sto}d^{comp,sto}_{n,sto}*STO^{in}_{n,sto,t}+\sum_{p}d^{comp,trans}_{n,p}*inc^{export}_{p,n}*\frac{F_{p,t}}{\eta^{trans}_{p}} \tag{16}$$

$$\ldots+\sum_{recon}G^{recon}_{n,recon,t} \tag{17}$$

The model endogenously determines electrolysis, storage, compression, and re-conversion capacity and operation. The operational decisions for hydrogen transport are also endogenous, while we assume exogenous transport capacities. Suppose $c^{var,sto}_{n,sto}$ are the operational costs for storage charging, $c^{var,recon}_{n,recon}$ the operational costs for re-conversion, c^{import}_{path} the costs for importing hydrogen from outside the modeled area. To avoid infeasibilities, we allow unspecified imports of hydrogen incurring the costs c^{infes} (here at prohibitively high 500 EUR per MWh_{ch}). Suppose further c^{oc}_{n} and c^{fix}_{n} are the overnight and fixed investment costs, we impose them on the capacity of electrolysis technology ely, compression capacity after electrolysis $comp, ely$, compression capacity for storage injection $comp, sto$, energy capacity of storage technology sto, and capacity of re-conversion technology $recon$. We add these operational (Supplementary Equation (18)) and investment costs (Supplementary Equation (19)) to the model's optimization function, which minimizes total system costs. The latter is documented in Zerrahn and Schill:[1]

$$\ldots+\sum_{n,sto,t}c^{var,sto}_{n,sto}*STO^{in}_{n,sto,t}+\sum_{n,recon,t}c^{var,recon}_{n,recon}*G^{recon}_{n,recon,t}+\sum_{path}c^{import}_{path}*IM_{path}+\sum_{n,t}c^{infes}*INFES_{n,t} \tag{18}$$

$$\begin{aligned}\ldots&+\sum_{n,ely}(c^{oc,ely}_{n,ely}+c^{fix,ely}_{n,ely})*N^{ely}_{n,ely}+\sum_{n,ely}(c^{oc,comp,ely}_{n,ely}+c^{fix,comp,ely}_{n,ely})*N^{comp,ely}_{n,ely}\\&+\sum_{n,sto}(c^{oc,comp,sto}_{n,sto}+c^{fix,comp,sto}_{n,sto})*N^{comp,sto}_{n,sto}+\sum_{n,sto}(c^{oc,sto}_{n,sto}+c^{fix,sto}_{n,sto})*N^{sto}_{n,sto}\\&+\sum_{n,recon}(c^{oc,recon}_{n,recon}+c^{fix,recon}_{n,recon})*N^{recon}_{n,recon}\end{aligned} \tag{19}$$

Supplementary Notes

Supplementary Note 1

Additional renewable drought illustrations: The duration and severity of most extreme winter droughts captured by the drought mass metric varies significantly across years and countries (Supplementary Fig. 1). Assuming perfect interconnection between all European countries, the most extreme event in the data occurred in the winter of 1996/97 and lasted 55 days. This European super drought was caused by pronounced and temporally overlapping events in many, yet not all, European countries (Figure 5). Hence, even during this extreme event, geographical balancing remains possible to a limited extent. It is therefore substantially shorter than the most extreme droughts in nearly all countries when considered an energy island. Applying the drought mass metric to individual countries, we find the longest winter events in Eastern and Southern Europe. Further, smaller countries such as Slovenia (182 days, 2013/14) or Slovakia (182 days, 2015/16) tend to have longer extreme droughts than larger countries such as France (64 days, 2004/05), Sweden (83 days, 1997/98), Germany (109 days, 1995/96), or Spain (131 days, 1988/89). This is because smaller countries benefit less from geographical balancing within their borders.

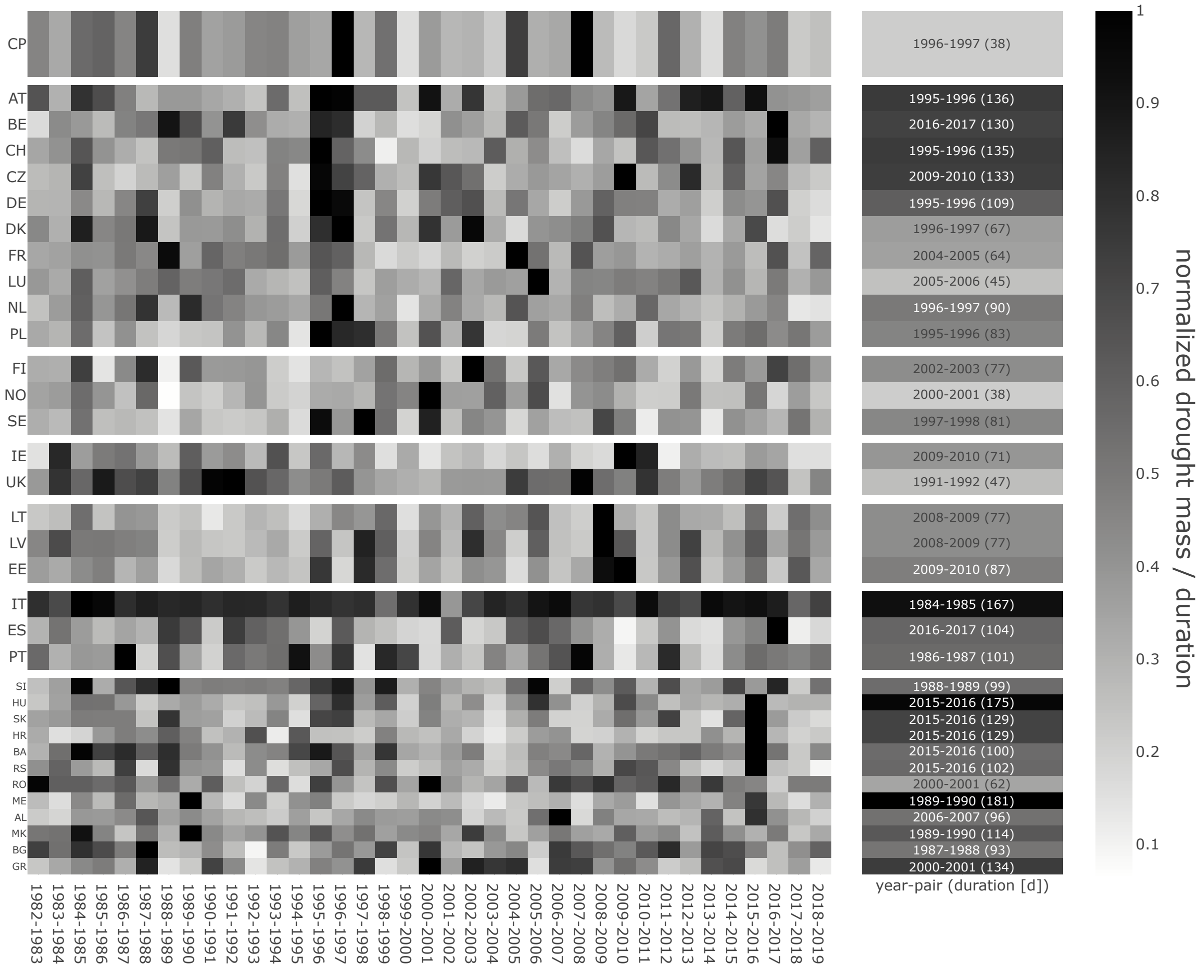

Supplementary Fig. 1: Drought mass of identified most extreme simulated winter drought events. For each country or the European copperplate (CP) in the left panel, drought mass scores are normalized using the row-specific maximum. The colors of the right panel indicate the maximum duration of the event with the highest drought mass score normalized by the column-specific maximum, i.e., the maximum duration across all countries. The right panel also provides the year-pair with the most severe events as identified by the drought mass score per row and its corresponding duration in days.

Supplementary Fig. 2 illustrates drought patterns of solar PV, onshore wind power and offshore wind power for three selected countries and the European copperplate for the year 1996/97. It can be seen that the largest droughts that emerge in renewable energy technology portfolios (panel a) are the result of coinciding drought events of individual technologies (panels b-d) in wintertime.

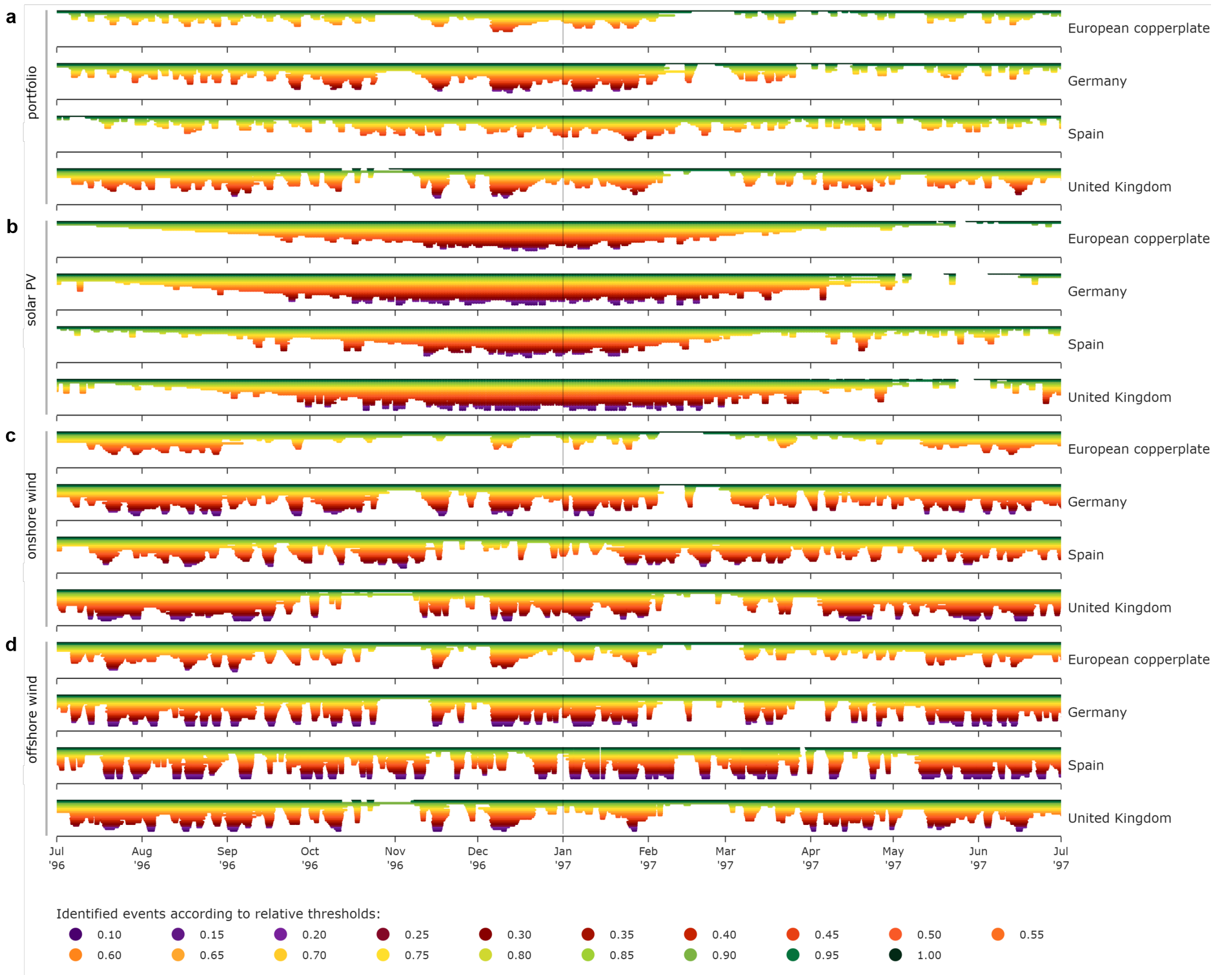

Supplementary Fig. 2: Simulated drought patterns in 1996 and 1997 for all investigated relative thresholds $\tau \in [0.1, 1]$ and selected regions. For each technology-specific panel, a horizontal band indicates drought occurrences for the color-coded threshold of one region. To illustrate persistent patterns, only droughts lasting longer than one day are displayed. Panel **a** corresponds to policy-oriented renewable technology portfolios, **b** to solar photovoltaics (PV), **c** to onshore wind, and **d** to offshore wind.

Supplementary Note 2

Additional insights on the correlation between renewable droughts and long-duration storage energy: Supplementary Fig. 3 compares the correlation between winter droughts and long-duration storage needs, considering either long-duration storage only, or hydro and long-duration storage technologies combined. Including the latter in the regression shows a substantial level effect for countries with low regression coefficients, i.e., low sensitivity of long-duration storage needs to increasingly severe droughts, visible by the upward shift of the respective regression lines in Supplementary Fig. 3b compared to Supplementary Fig. 3b. This indicates that mid-term flexibility options can substitute long-duration storage needs for dealing with extreme droughts to a significant extent.

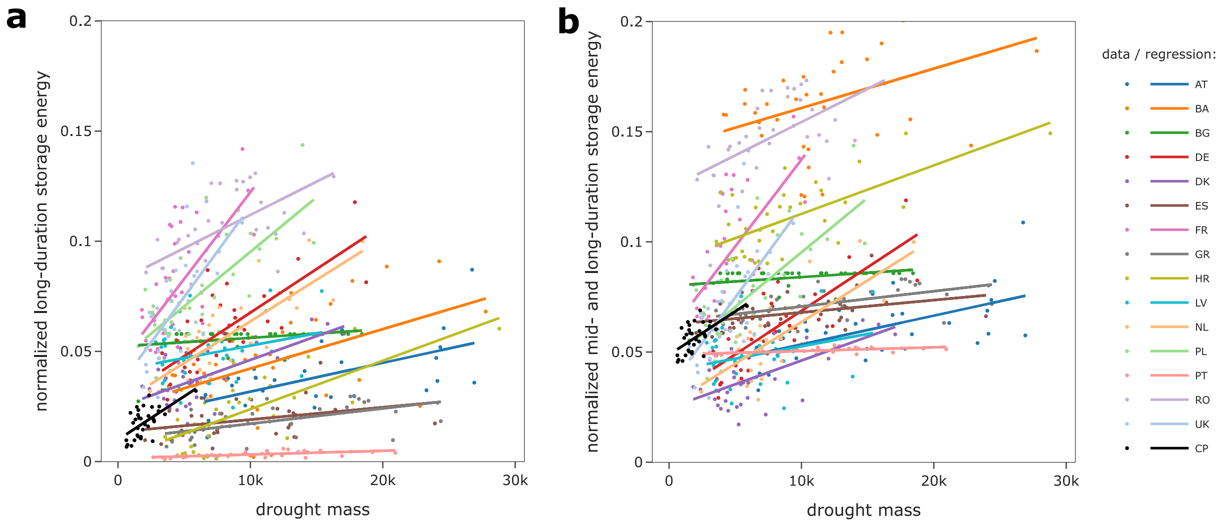

Supplementary Fig. 3: Correlation of winter drought mass and different types of storage energy normalized by annual demand for electricity across years. For comparison, we normalize the least-cost storage energy by annual demand for electricity (including electrified heating). For illustration, we exclude countries with negligible storage energy or storage energy at potential but include the pan-European copperplate scenario (CP). **a** Long-duration storage only. **b** Mid- and long-duration storage.

Supplementary Fig. 4 shows additional regression results for a complementary Germany-only setting using flat electricity demand profiles to exemplarily disentangle the storage-defining effect of renewable droughts from the storage-driving effect of demand seasonality (compare blue and orange lines in Supplementary Fig. 4). The slope of the regression line decreases with flatter electricity demand profiles, i.e., the storage-defining effect of droughts is less pronounced without demand seasonality.

In an additional sensitivity analysis, we assess how an alternative capacity mix of wind and solar PV across Europe affects drought mass results and how this relates to least-cost long-duration storage outcomes. We compare the correlation between long-duration storage energy and drought mass values for “Distributed Energy” (default) and the “Global Ambition” scenarios of the TYNDP 2022 (Supplementary Fig. 5). The “Distributed Energy” scenario features more solar PV, while the “Global Ambition” scenario includes a higher share of offshore wind. In the latter, the drought mass of the most extreme events tends to decrease in some countries and the copperplate scenario. This is due to the lower solar PV share, which alleviates the impact of solar seasonality on compound portfolio droughts in winter. However, regression slopes hardly change, indicating a limited impact of the alternative capacity mix on drought mass results. The most extreme drought for the copperplate scenario occurs in both scenarios in the winter of 1996/97.

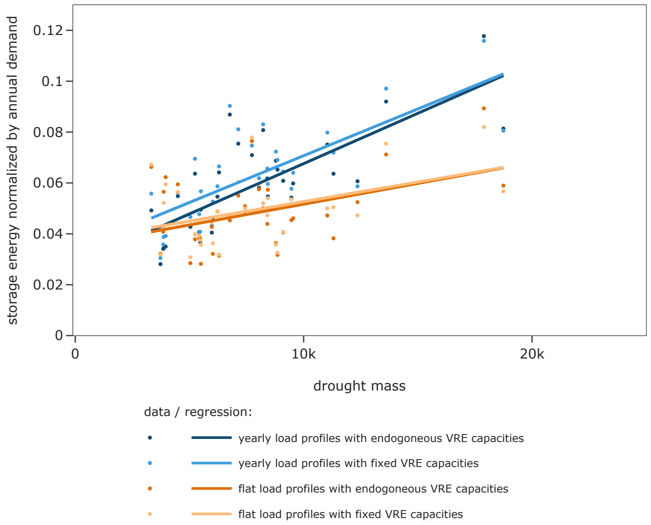

Supplementary Fig. 4: Correlation of the drought mass of most extreme winter drought events and normalized storage energy capacity in Germany. For comparison, we normalize the least-cost storage energy by annual demand for electricity (including electrified heating). The renewable portfolio assumptions of the scenarios with fixed VRE capacities align with those used for the time series-based VRE drought analysis. In contrast, the scenarios with endogenous VRE capacities are optimized by our power sector model.

The scatter plots in Figure 2 indicate that the correlation between the most extreme winter drought event and the least-cost long-duration storage size for the same year is not perfect. Several factors explain this. First, country-specific factors such as varying portfolios of variable or firm renewable generation capacity or flexibility options, e.g., high shares of reservoir power plants in the Spanish capacity mix, may cause the imperfect fit. Second, demand peaks vary substantially between weather years in terms of level and temporal variation, especially in winter (compare France in Figure 3). This means that similar droughts can trigger different storage needs, depending on the load situation. Third, our measurement of drought events is, by design, purely based on renewable availability time series and does not consider pre- or succeeding periods of very high availability or the seasonality of electricity demand. In contrast, the power sector optimization factors in these aspects. In addition, the drought mass metric based on the VMBT method relies on the choice of a cut-off threshold and is solely an approximation of the cumulative energy deficit of drought events,[2] which is relevant for long-duration storage needs. Due to the averaging mechanism of the VMBT method, this metric tends to underestimate solar PV contributions within extreme droughts, particularly in countries with a less pronounced solar seasonality. These contributions generally lead to higher deployment of short-duration system flexibility and lower long-duration storage needs. Finally, VRE portfolios differ to some extent between the drought analysis and the power sector model. While these portfolios are fixed in the former, they are endogenously optimized in the latter, yielding slightly different capacity mixes between weather years. Yet, the overall fit between the indicators appears to be reasonable, which

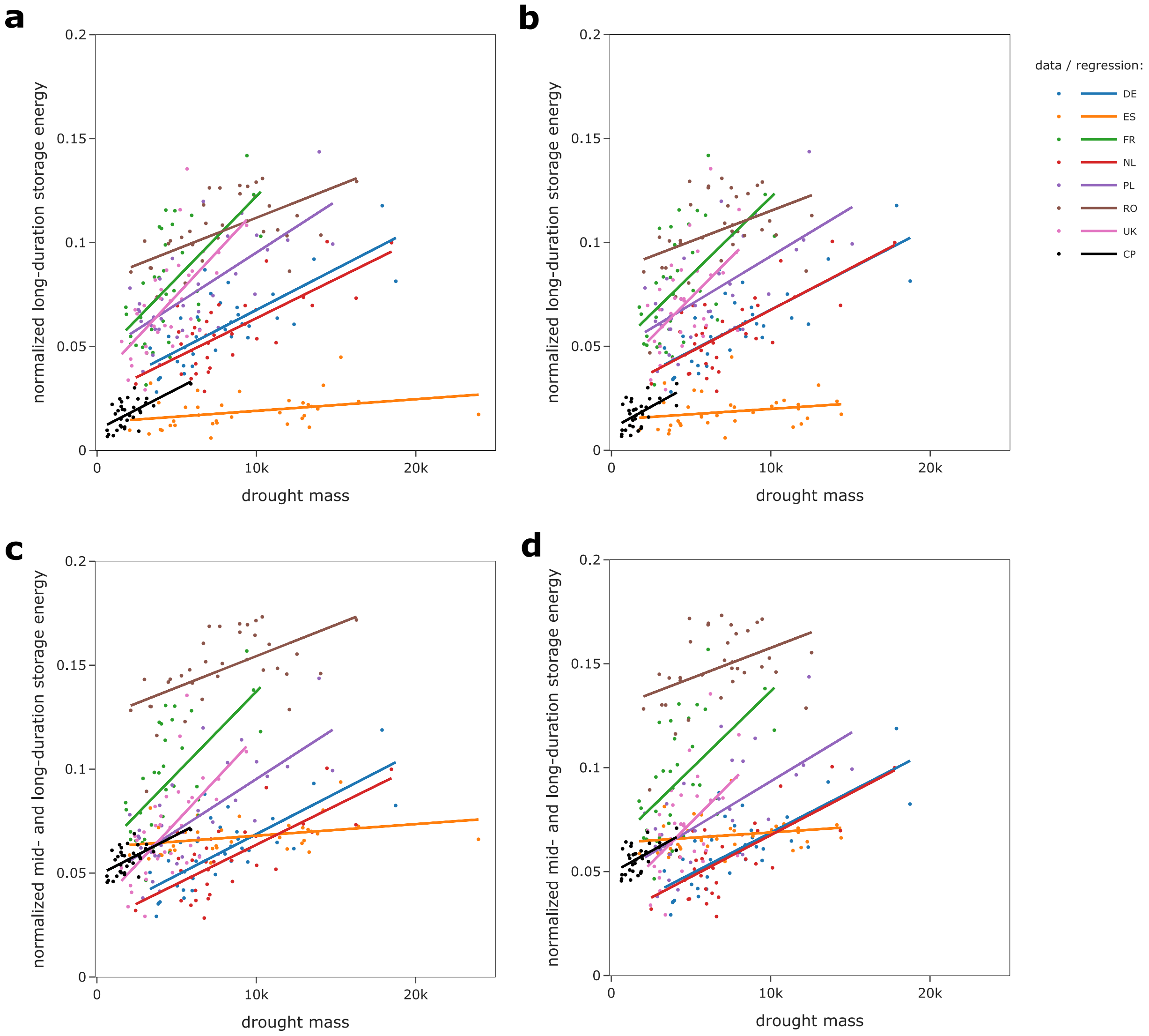

Supplementary Fig. 5: Correlation of the drought mass of most extreme winter drought events and normalized storage energy capacity. The illustrations on the left-hand side represent our default setting, which uses the wind and solar PV capacity mix of the TYNDP 2022 "Distributed Energy" scenario for drought mass computation. The regressions on the right-hand side are based on wind and solar capacity assumptions from the TYNDP 2022 "Global Ambition" scenario. For comparison, we normalize the least-cost storage energy with the annual demand for electricity (including electrified heating). For illustration, we exclude countries with least-cost storage energy below 5 TWh or investment at potential but include the pan-European copperplate scenario (CP). **a** Long-duration storage only (default). **b** Long-duration storage only (alternative capacity mix). **c** Mid- and long-duration storage (default). **d** Mid- and long-duration storage (alternative capacity mix).

can also be confirmed by a complementary Germany-only analysis (compare dark and bright lines in Supplementary Fig. 4).

Supplementary Note 3

Additional illustrations of the impact of interconnection on long-duration storage energy: The weather years 1987/88 and 1988/89 exhibit similar long-duration energy storage capacities for the energy island scenario (Figure 4). For increasing interconnection levels, least-cost storage capacity diverges, yielding substantially lower energy storage levels for 1988/89 compared to 1987/88. This is because the most pronounced renewable droughts are temporally highly correlated with each other and, to some extent, also with high-demand periods in 1987/88 across many countries. In contrast, this is not the case in 1988/89 (compare boxes in Supplementary Fig. 6 and Supplementary Fig. 7), enabling more pronounced geographical balancing of these drought events. The temporal overlap is even more pronounced in weather year 1996/97 (Figure 5), which leads to the most extreme pan-European drought in the data (Supplementary Fig. 1) and, accordingly, the highest long-duration storage needs (Figure 4). Supplementary Fig. 9, Supplementary Fig. 10, and Supplementary Fig. 11 show the impact of additional nuclear power on the least-cost deployment of solar PV, onshore wind, and offshore wind. Note that in the default setting with renewable generation only, which bases on the assumptions of the "Distributed Energy" scenario of the TYNDP 2022,[3] we adjusted the expansion bounds for variable renewable technologies accounting for the missing generation of nuclear power. This results in slightly increased lower expansion bounds compared to the complementary runs including nuclear power, which are binding for PV and offshore wind in scenario (4).

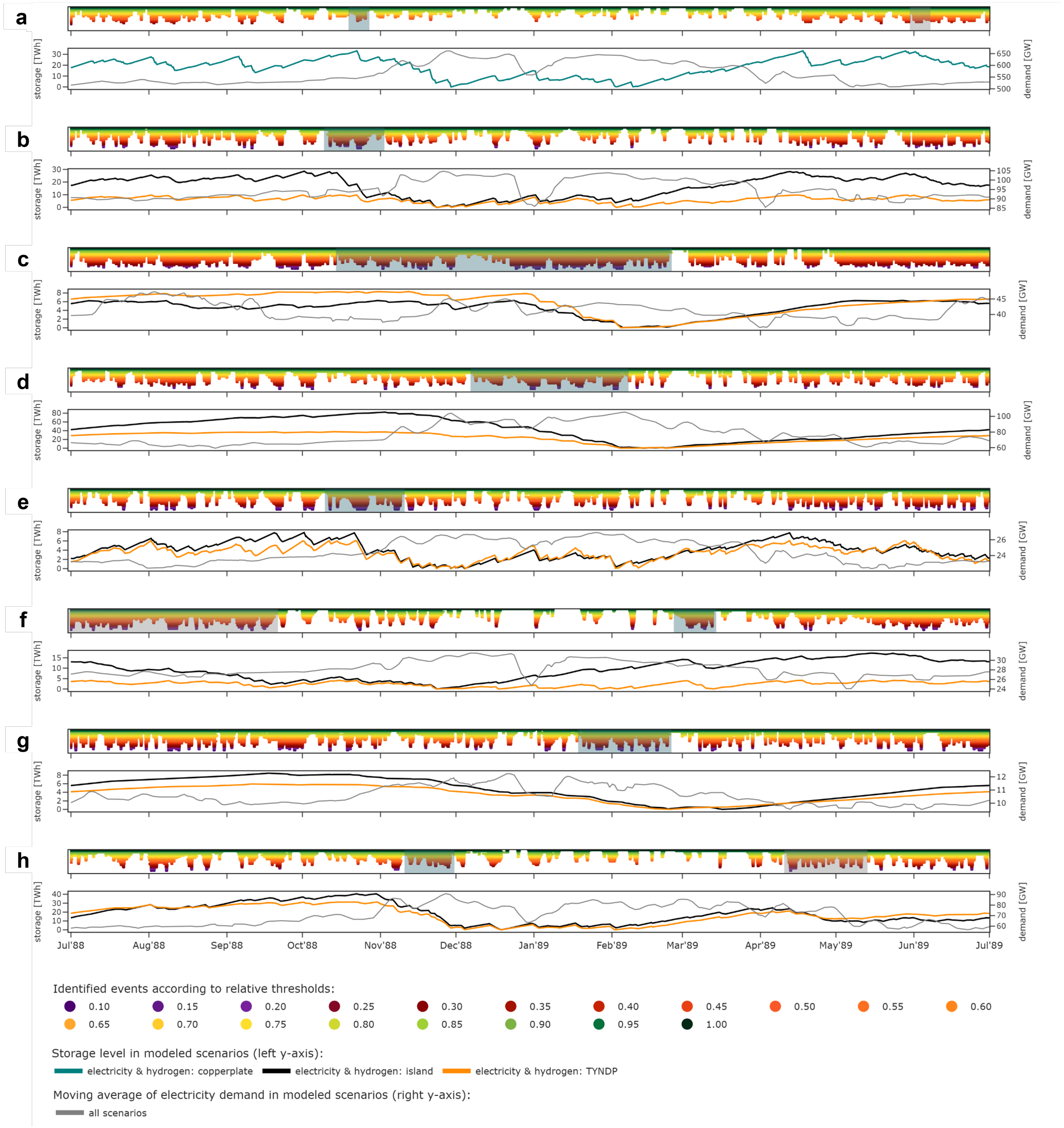

Supplementary Fig. 6: Simulated drought events, electricity demand, and least-cost state-of-charge of long-duration storage in winter 1988/89 in countries with highest long-duration storage energy capacities. The top row of each panel shows the identified drought patterns lasting longer than 12 hours across all color-coded thresholds, with the most extreme drought events occurring in winter (teal boxes) or throughout the year (gray boxes). The bottom row of each panel displays the associated exogenous smoothed demand profiles used in the optimization and the resulting least-cost storage state-of-charge levels for isolated countries modeled within the interconnection scenario (1), for policy-oriented interconnection levels in scenario (3), or the pan-European copperplate in scenario (4). Panel **a** corresponds to the pan-European copperplate scenario, **b** to Germany, **c** to Spain, **d** to France, **e** to the Netherlands, **f** to Poland, **g** to Romania, and **h** to the United Kingdom.

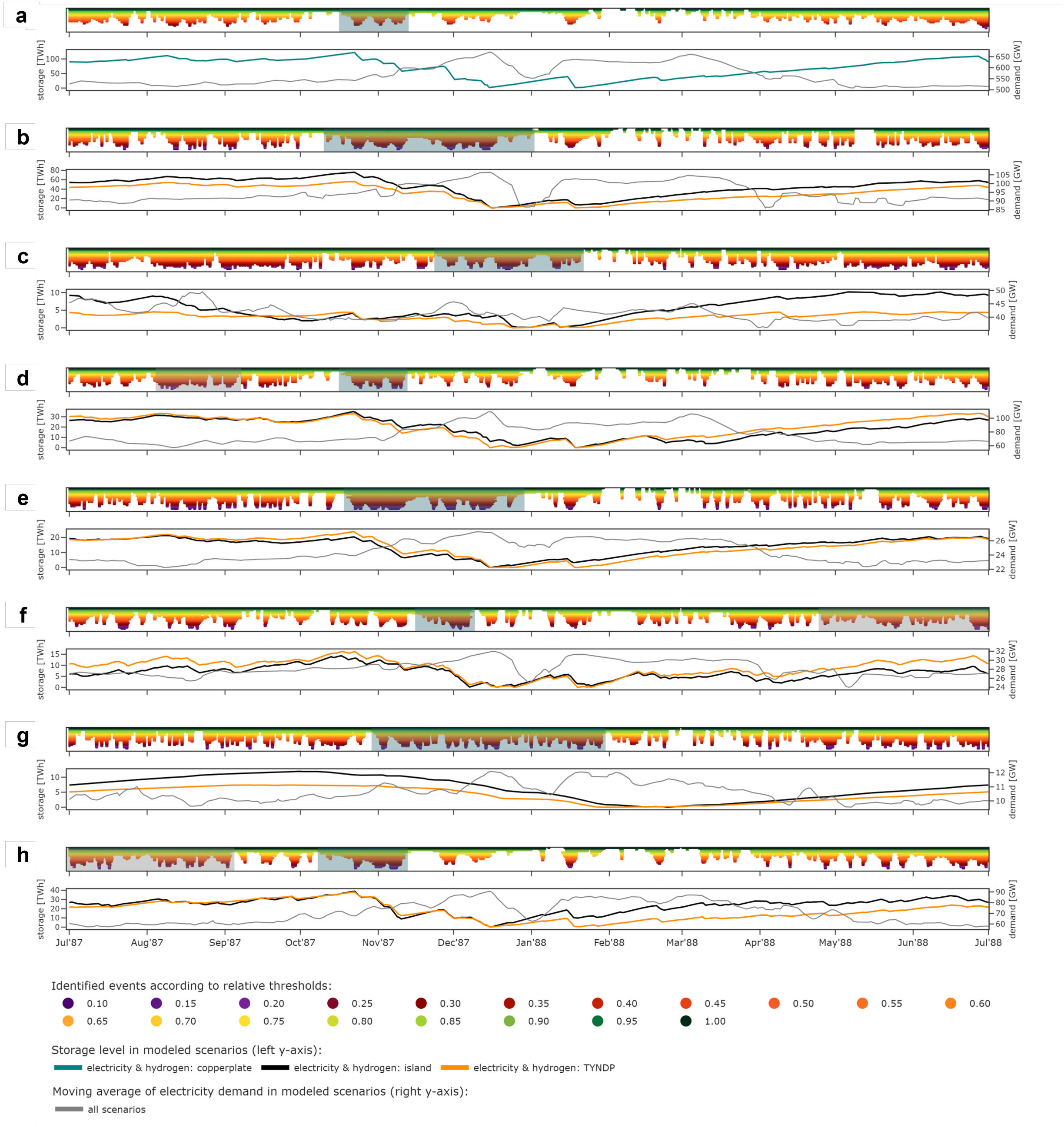

Supplementary Fig. 7: Simulated drought events, electricity demand, and least-cost state-of-charge of long-duration storage in winter 1987/88 in countries with highest long-duration storage energy capacities. The top row of each panel shows the identified drought patterns lasting longer than 12 hours across all color-coded thresholds, with the most extreme drought events occurring in winter (teal boxes) or throughout the year (gray boxes). The bottom row of each panel displays the associated exogenous smoothed demand profiles used in the optimization and the resulting least-cost storage state-of-charge levels for isolated countries modeled within the interconnection scenario (1), for policy-oriented interconnection levels in scenario (3), or the pan-European copperplate in scenario (4). Panel **a** corresponds to the pan-European copperplate scenario, **b** to Germany, **c** to Spain, **d** to France, **e** to the Netherlands, **f** to Poland, **g** to Romania, and **h** to the United Kingdom.

Generally, interconnection mitigates long-duration storage energy needs (Figure 4). Overall, the

ranking of weather years is relatively persistent, yet the storage-mitigating effect varies between weather years (Supplementary Fig. 8), especially for higher levels of interconnection. The mechanisms behind this effect, in particular temporally correlated severe drought events, are illustrated above.

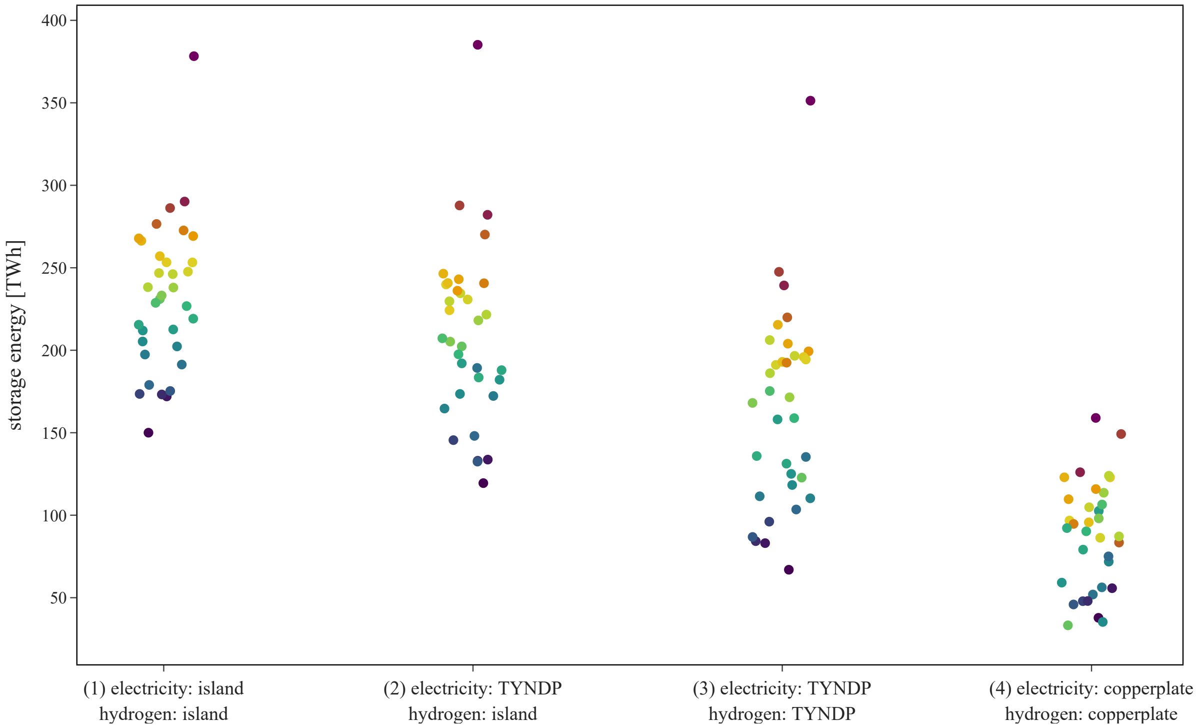

Supplementary Fig. 8: Long-duration storage energy capacity aggregated across all countries for all modeled interconnection scenarios. Every point refers to a single weather year. The coloring changes continuously in long-duration storage energy capacity according to the ranking of weather years in scenario (1). The weather year color remains consistent across the other scenarios.

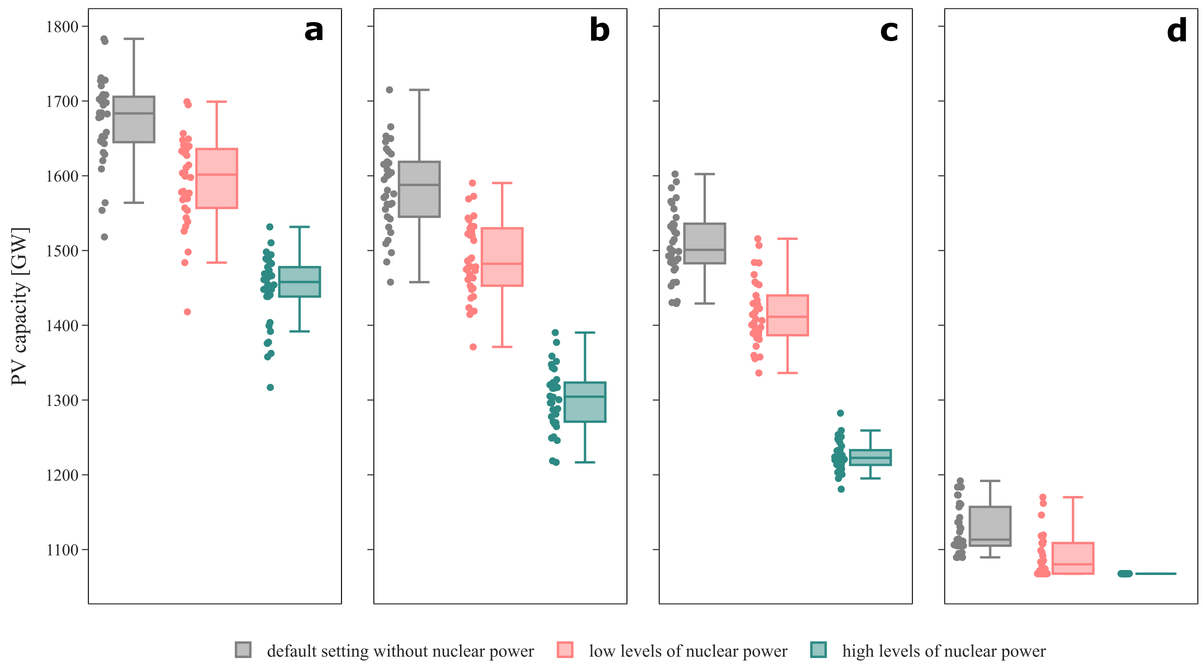

Supplementary Fig. 9: Least-cost solar PV capacity aggregated across all countries for all modeled weather years and interconnection scenarios. Each dot refers to one independently modeled weather year. The center line denotes the median, box limits indicate the interquartile range (Q1–Q3), whiskers extend to 1.5× the interquartile range, and points beyond the whiskers represent outliers. **a** Scenario (1): no exchange of electricity nor hydrogen (island systems). **b** Scenario (2): policy-oriented exchange of electricity, no exchange of hydrogen. **c** Scenario (3): policy-oriented exchange of electricity and hydrogen. **d** Scenario (4): pan-European copperplate assuming unconstrained exchange of electricity and hydrogen.

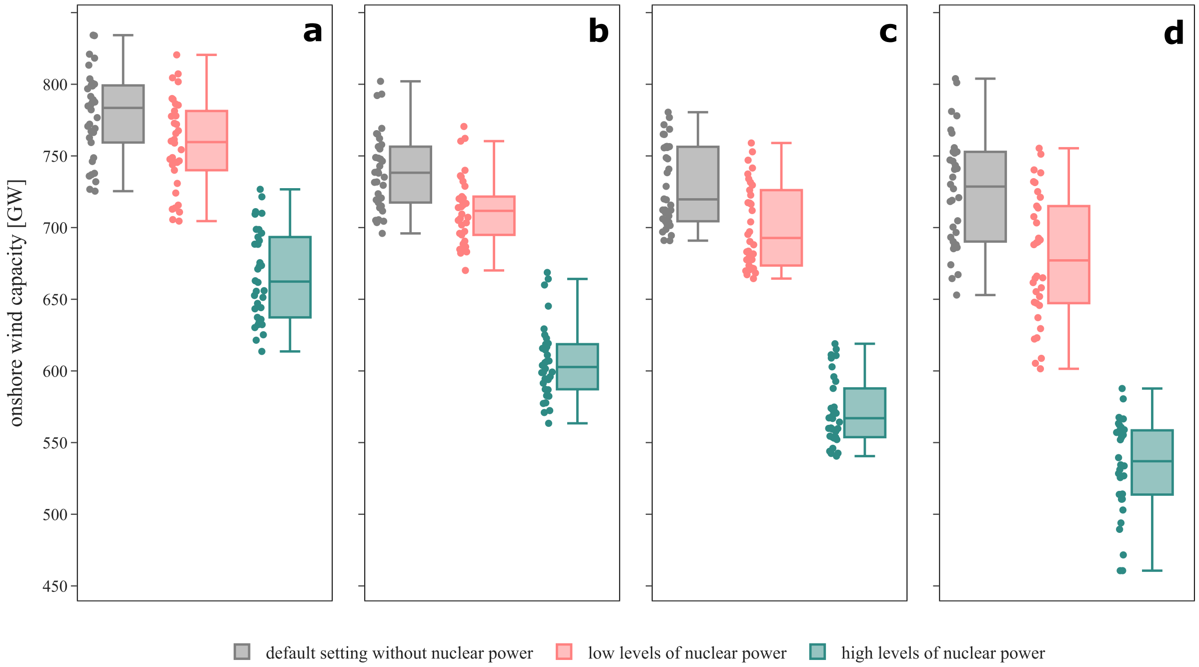

Supplementary Fig. 10: Least-cost onshore wind capacity aggregated across all countries for all modeled weather years and interconnection scenarios. Each dot refers to one independently modeled weather year. The center line denotes the median, box limits indicate the interquartile range (Q1–Q3), whiskers extend to 1.5× the interquartile range, and points beyond the whiskers represent outliers. **a** Scenario (1): no exchange of electricity nor hydrogen (island systems). **b** Scenario (2): policy-oriented exchange of electricity, no exchange of hydrogen. **c** Scenario (3): policy-oriented exchange of electricity and hydrogen. **d** Scenario (4): pan-European copperplate assuming unconstrained exchange of electricity and hydrogen.

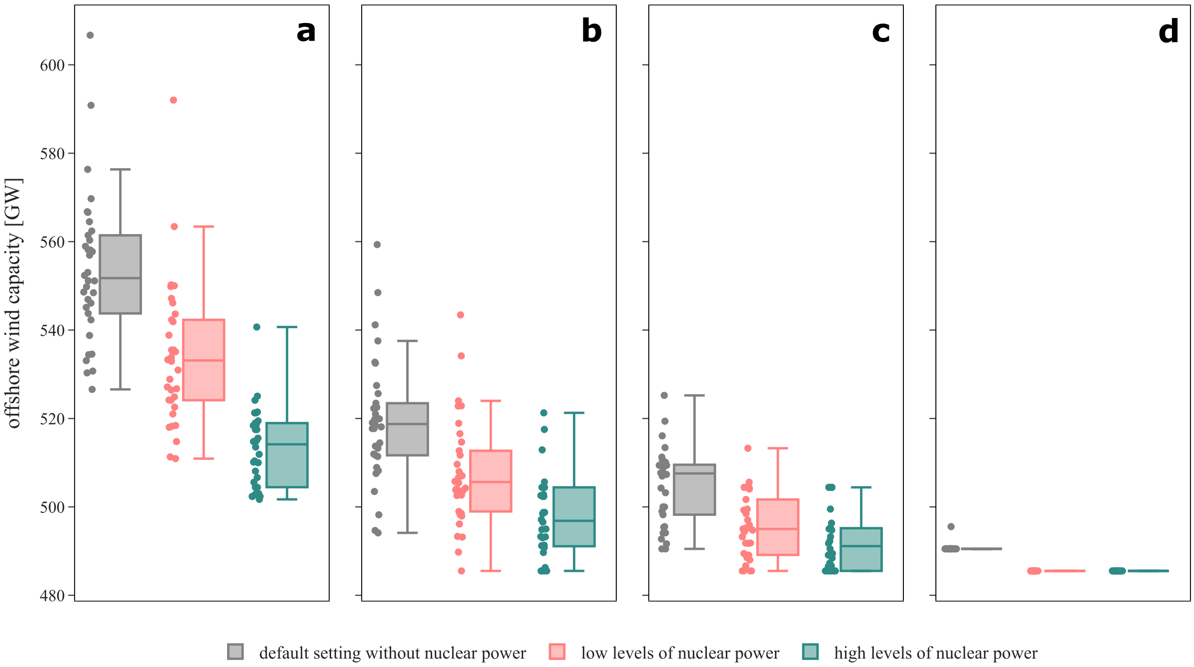

Supplementary Fig. 11: Least-cost offshore wind capacity aggregated across all countries for all modeled weather years and interconnection scenarios. Each dot refers to one independently modeled weather year. The center line denotes the median, box limits indicate the interquartile range (Q1–Q3), whiskers extend to 1.5× the interquartile range, and points beyond the whiskers represent outliers. **a** Scenario (1): no exchange of electricity nor hydrogen (island systems). **b** Scenario (2): policy-oriented exchange of electricity, no exchange of hydrogen. **c** Scenario (3): policy-oriented exchange of electricity and hydrogen. **d** Scenario (4): pan-European copperplate assuming unconstrained exchange of electricity and hydrogen.

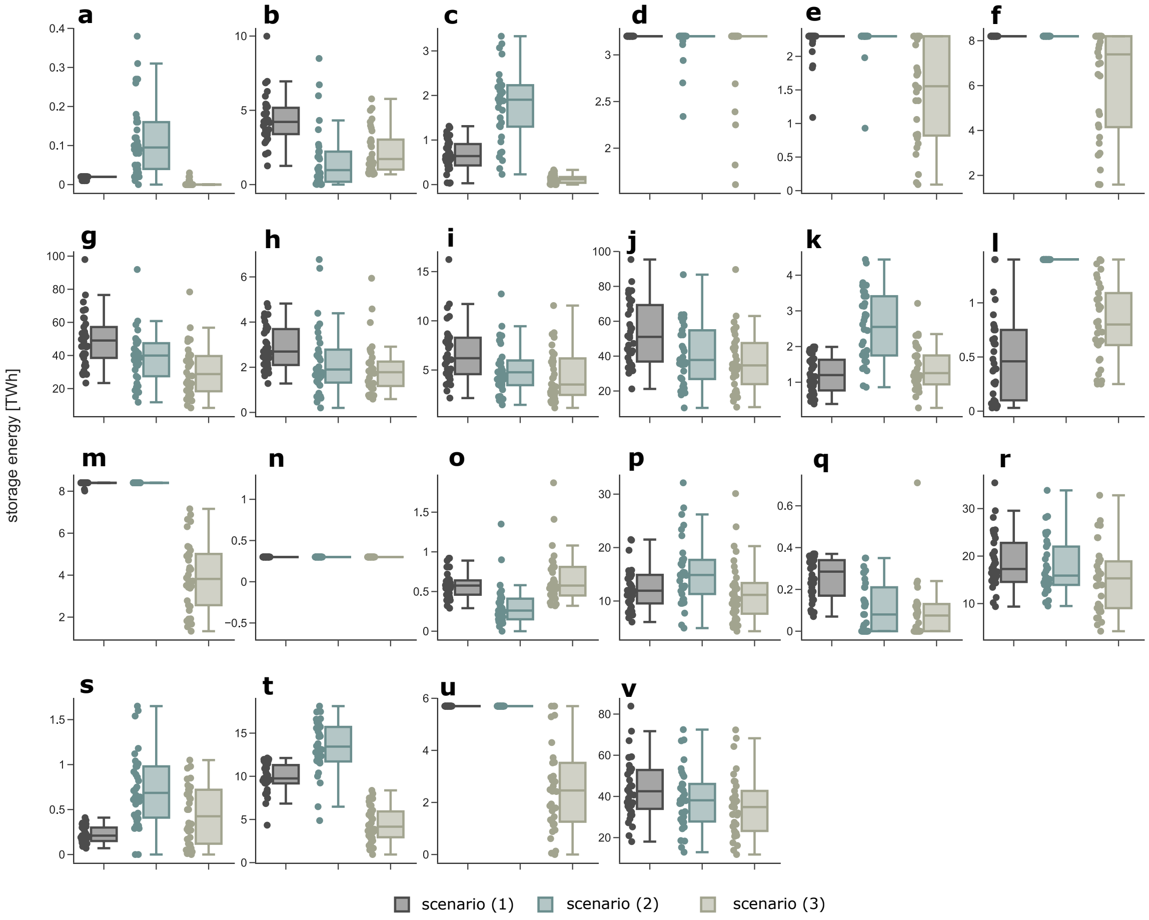

Supplementary Fig. 12: Long-duration storage energy capacities across all countries with storage potential across all weather years and interconnection scenarios. Each dot refers to one independently modeled weather year. The center line denotes the median, box limits indicate the interquartile range (Q1–Q3), whiskers extend to 1.5× the interquartile range, and points beyond the whiskers represent outliers. Note that the vertical axis are scaled for each country for illustration. Many of the effects in countries with smaller power sectors are minor relative to the aggregated effects discussed in the main body of this work. The pan-European copperplate scenario is omitted as the geographical distribution of long-duration storage capacities is arbitrary and hence results cannot be meaningful interpreted. **a** Algeria. **b** Austria. **c** Bosnia-Herzegovina. **d** Belgium. **e** Bulgaria. **f** Czech Republic. **g** Germany. **h** Denmark. **i** Spain. **j** France. **k** Greece. **l** Croatia. **m** Hungary. **n** Italy. **o** Latvia. **p** the Netherlands. **q** Norway. **r** Poland. **s** Portugal. **t** Romania. **u** Slovakia. **v** the United Kingdom.

Increasing the interconnection capacities decreases the need for long-duration storage energy capacities (Figure 4) on a aggregate European level, but also individually in most countries (Supplementary Fig. 12).

Supplementary Note 4

Additional information on the impact of firm zero-emission generation: Supplementary Fig. 13 and Supplementary Fig. 14 visualizes the hourly and daily nuclear generation aggregated across all countries in the winter of 1996/97 across all interconnection scenarios for low and

high levels of nuclear power. Supplementary Fig. 15 and Supplementary Fig. 16 show the hourly operation of nuclear capacity aggregated across all countries for all interconnection scenarios and weather years for low and high levels of nuclear power.

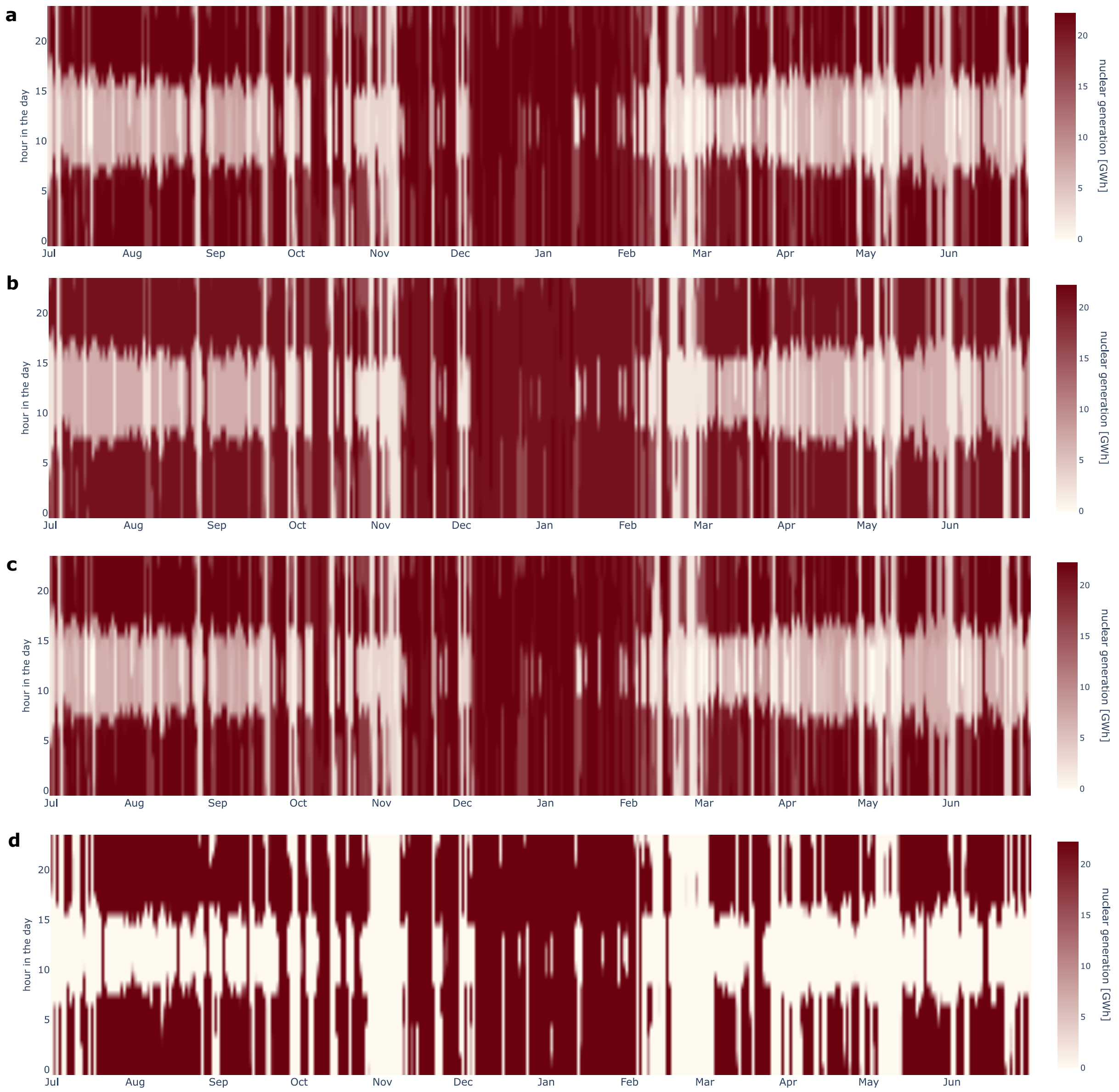

Supplementary Fig. 13: Hourly and daily generation patterns of nuclear power aggregated across all countries in 1996/97 in the scenario with low levels of nuclear power. **a** Scenario (1): no exchange of electricity or hydrogen. **b** Scenario (2): policy-oriented exchange of electricity. **c** Scenario (3): policy-oriented exchange of electricity and hydrogen. **d** Scenario (4): unconstrained exchange of electricity or hydrogen.

While limited in near-term scalability,[4] other firm zero-emission generation technologies may become viable in the longer run, such as advanced nuclear fission or fusion or advanced geothermal power generation. Such technologies are expected to have very high capital costs but low operational costs, which implies that they would optimally operate at very high full-load hours.[5]

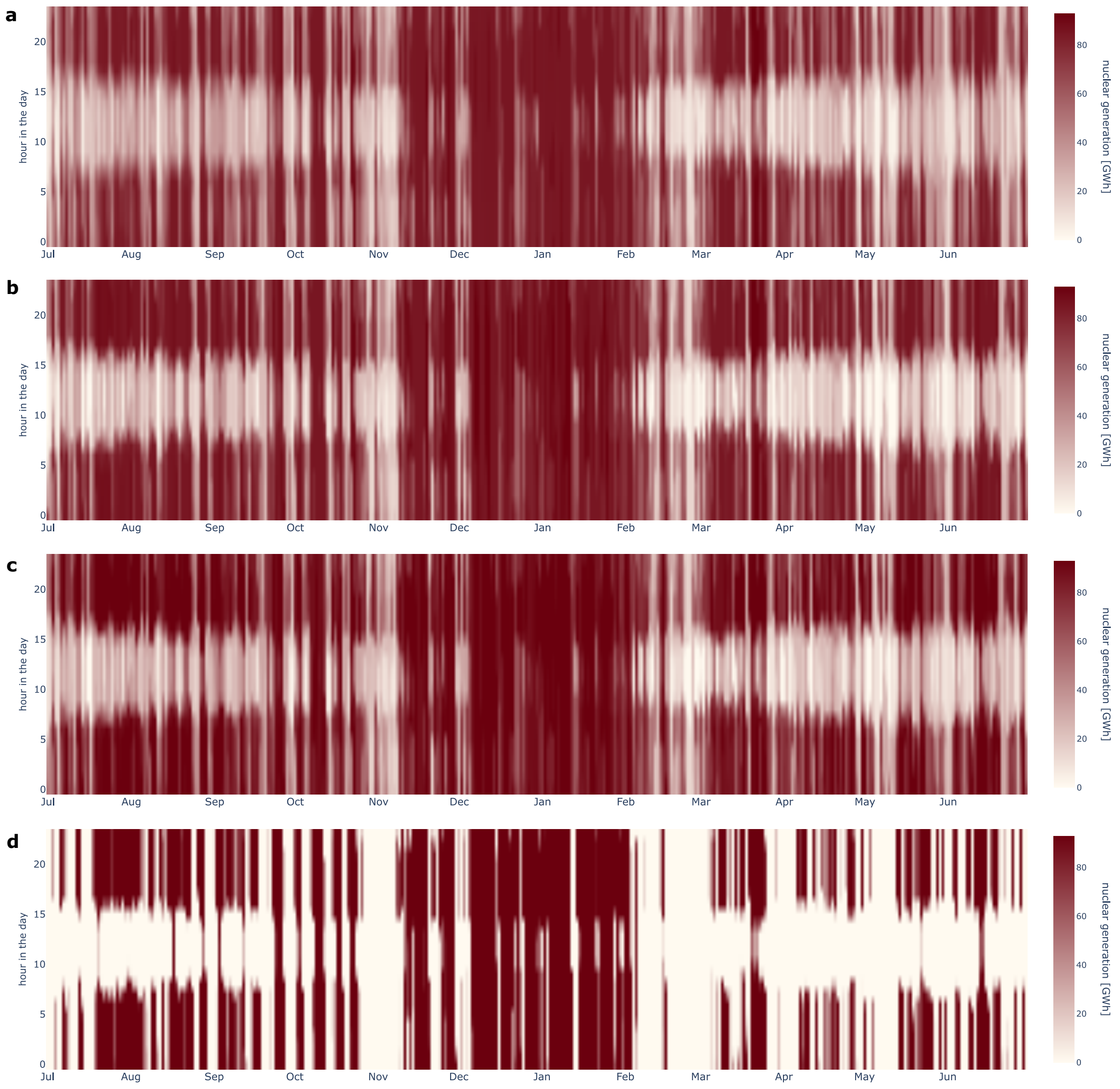

Supplementary Fig. 14: Hourly and daily generation patterns of nuclear power aggregated across all countries in 1996/97 in the scenario with high levels of nuclear power. **a** Scenario (1): no exchange of electricity or hydrogen. **b** Scenario (2): policy-oriented exchange of electricity. **c** Scenario (3): policy-oriented exchange of electricity and hydrogen. **d** Scenario (4): unconstrained exchange of electricity or hydrogen.

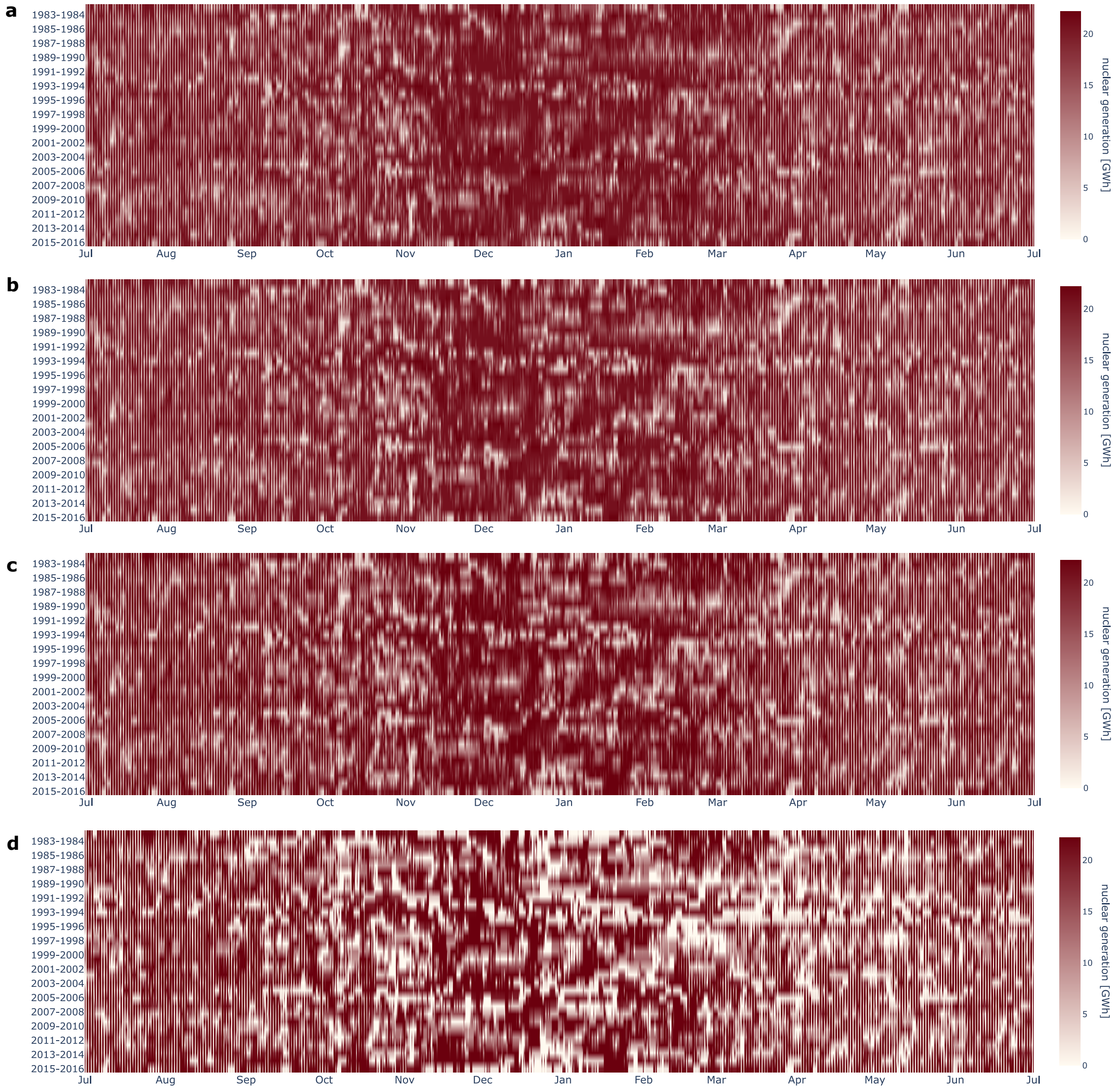

Supplementary Fig. 15: Hourly and yearly generation patterns of nuclear power aggregated across all countries for all weather years in the scenarios with low levels of nuclear power. **a** Scenario (1): no exchange of electricity or hydrogen. **b** Scenario (2): policy-oriented exchange of electricity. **c** Scenario (3): policy-oriented exchange of electricity and hydrogen. **d** Scenario (4): unconstrained exchange of electricity or hydrogen.

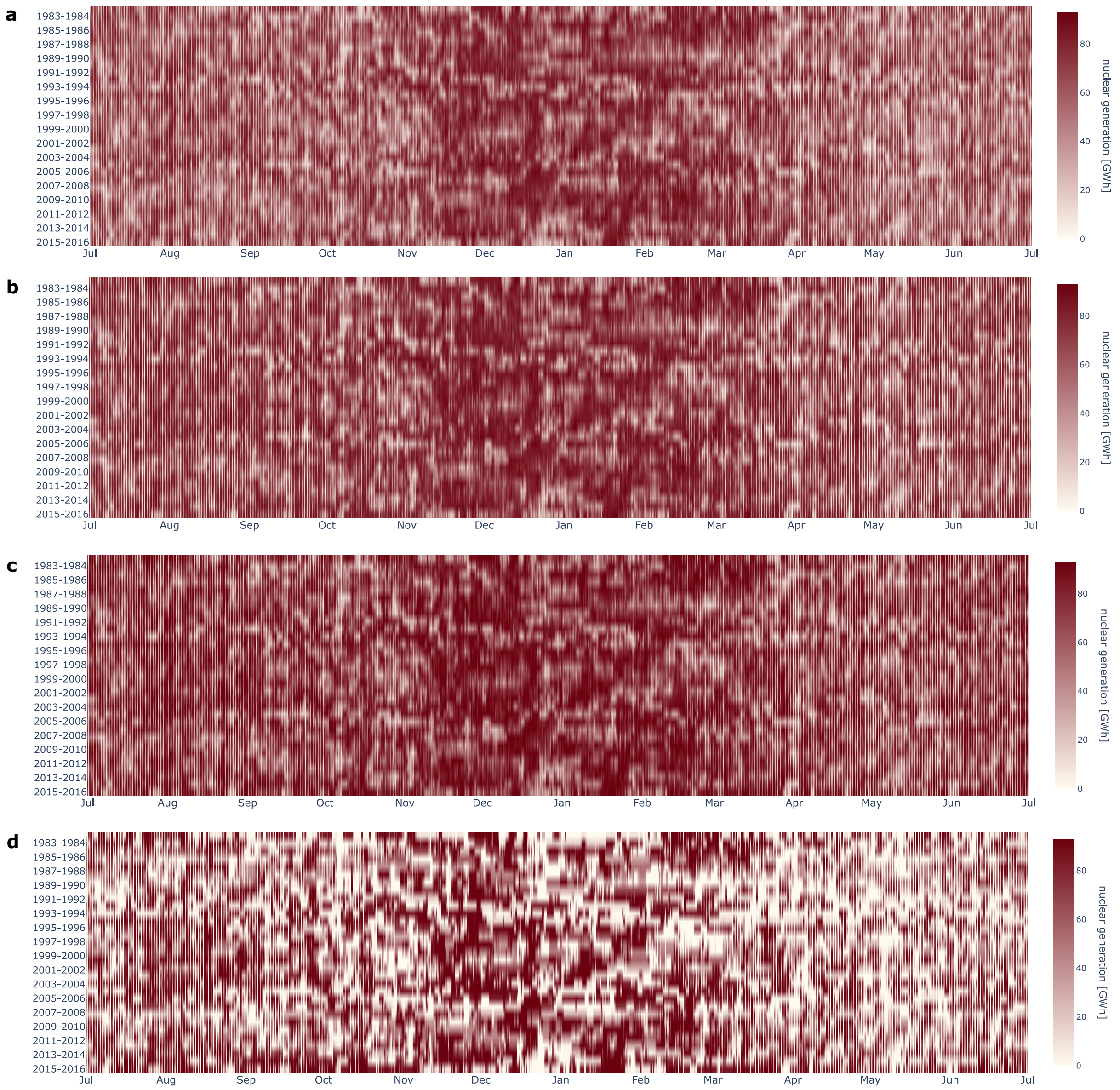

Supplementary Fig. 16: Hourly and yearly generation patterns of nuclear power aggregated across all countries for all weather years in the scenarios with high levels of nuclear power. **a** Scenario (1): no exchange of electricity or hydrogen. **b** Scenario (2): policy-oriented exchange of electricity. **c** Scenario (3): policy-oriented exchange of electricity and hydrogen. **d** Scenario (4): unconstrained exchange of electricity or hydrogen

Based on the weather year 1996/97, which includes a very pronounced renewable drought in many European countries, we analyze the impact of a generic dispatchable zero-emission technology on long-duration storage for the illustrative example of Germany, modeled as an energy island to limit the computation burden. In a series of 51 model runs, we iteratively increase the exogenous generation capacity of the zero-emission capacity by 1 GW increments as dispatch and investment decisions of all other generation and storage technologies remain endogenous. The firm zero-emission technology can continuously generate electricity not only during extreme droughts but also throughout the entire modeled weather year. This reduces the reliance on variable wind and solar power, which decreases the need for system flexibility.

Supplementary Fig. 17 illustrates these substitution effects. In addition to disproportionally displacing VRE capacity due to higher full-load hours, the increasing zero-emission generation capacity also reduces the need for battery storage, hydrogen gas turbines, and long-duration storage energy capacity. Yet, long-duration storage remains required as long as variable renewables are still part of the energy mix. This holds true even for very high levels of generation capacity of the zero-emission technology, going far beyond the peak nuclear power capacity ever reached in Germany.

The figure indicates a near-linear reduction of long-duration storage capacity for increasing levels of zero-emission generation capacity. Varying ratios of VRE technologies in the capacity mixes explain irregularities in the negative slope. For instance, for a firm zero-emission capacity of more than 43 GW, the model abstains from deploying cost-intensive offshore wind power. Each additional gigawatt of the zero-emission technology has now a significantly higher substitution rate to onshore wind and solar PV compared to scenarios with less capacity. This is because of the difference in full-load hours of these VRE technologies. In Germany, offshore wind has typically around twice the full-load hours of onshore wind and four times as many as PV. In scenarios without offshore wind, additional dispatchable capacity therefore replaces much more onshore wind and PV, which causes a more pronounced decrease in long-duration energy storage capacity.

Supplementary Note 5

Additional information on a sensitivity with varying values of lost load: Another option for dealing with extreme renewable droughts could be load shedding by system operators. This is considered as a last resort, as it can have substantial economic and societal implications. The value of lost load metric is often used to approximate the socio-economic costs of unmet electricity demand. Leaning on estimates by Kachirayil et al.,[6] Supplementary Fig. 18 shows the impact load shedding at values of lost load ranging between 1,000 and 20,000 EUR per MWh for the interconnection scenario (3) using the weather year 1996/97. At lower values of lost load, load shedding mainly displaces long-duration discharging capacity and shorter-duration flexibility such as

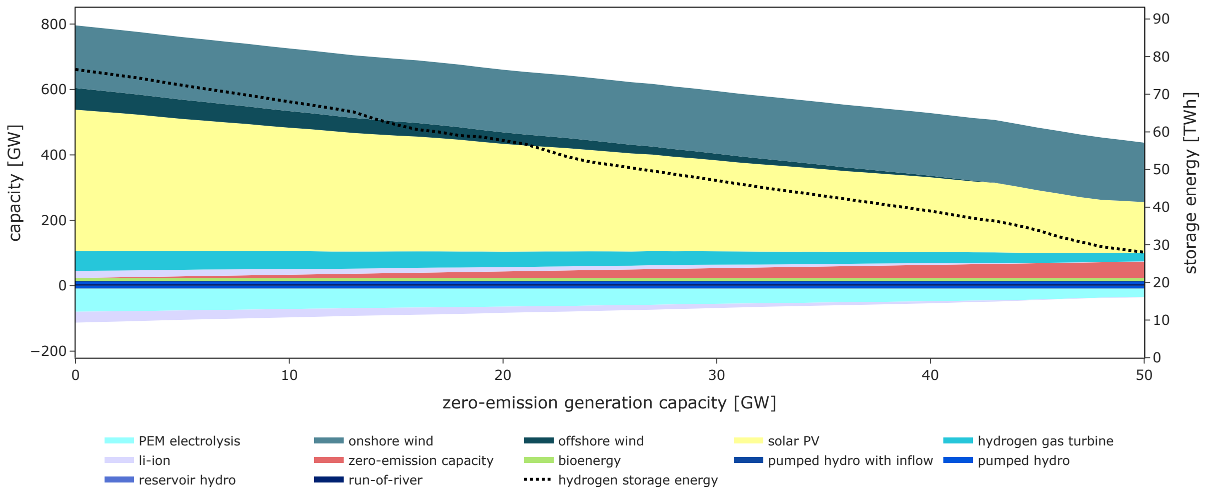

Supplementary Fig. 17: Least-cost wind and solar capacity as well as short- and long-duration flexibility options for increasing zero-emission generation capacity in a Germany only setting. The positive part of the left y-axis relates to generation and storage discharge, and its negative part to storage charge. The right y-axis refers to the long-duration storage energy. The expansion of solar PV as well as on- and offshore wind is limited according to the upper bounds of the TYNDP 2024, while we remove the lower expansion bounds.

bioenergy or batteries. This effect diminishes for higher, and more plausible, values of lost load. Least-cost long-duration storage energy capacity and total systems costs prove to be very robust. They decrease between zero and 1%, i.e., they are hardly affected by load shedding.

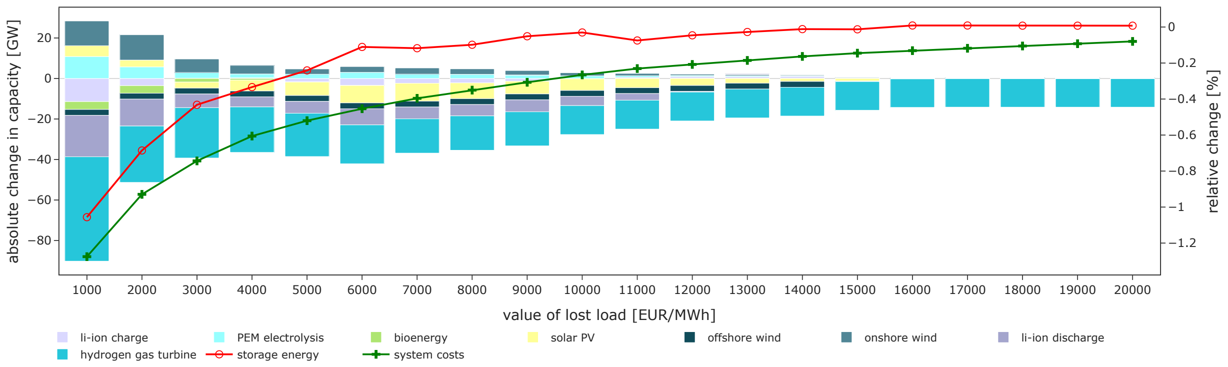

Supplementary Fig. 18: Change in capacities and total system costs for varying assumptions on the value of lost load. Absolute changes in capacities (bars, left y-axis) and relative change of long-duration energy storage capacity and total system costs (lines, right y-axis) aggregated across all countries in Europe in the interconnection scenario (3) compared to the default setting in 1996/97 for varying assumptions on the value of lost load. For readability, we show the zero line of the left y-axis in gray.

Supplementary Tables

Additional information on the cost sensitivity analysis

Supplementary Table 1: Overnight investment costs in the default setting as well as the lower and upper end of the cost range investigated in sensitivity analyses.

	default	variation range	lowest costs	highest costs
Solar PV [EUR/MW$_{el}$]	305,600	± 50%	152,800	458,400
Onshore wind [EUR/MW$_{el}$]	920,000	± 50%	460,000	1,380,000
Offshore wind [EUR/MW$_{el}$]	2,048,760	± 50%	1,024,380	3,073,140
Long-duration storage [EUR/MWh$_{th}$]	1,276	-50%, +400%	638	5,104

Supplementary References

[1] Zerrahn, A. & Schill, W.-P. Long-run power storage requirements for high shares of renewables: review and a new model. *Renewable and Sustainable Energy Reviews* **79**, 1518–1534 (2017). URL http://www.sciencedirect.com/science/article/pii/S1364032116308619.

[2] Kittel, M. & Schill, W.-P. Measuring the Dunkelflaute: how (not) to analyze variable renewable energy shortage. *Environmental Research: Energy* **1**, 035007 (2024). URL https://doi.org/10.1088/2753-3751/ad6dfc.

[3] ENTSO-e & ENTSO-g. TYNDP2022 Scenario Report. Tech. Rep. Version April 2022, European Network of Transmission System Operators for Electricity and Gas (2022).

[4] Calvin, K. *et al.* IPCC, 2023: Climate Change 2023: Synthesis Report. Contribution of Working Groups I, II and III to the Sixth Assessment Report of the Intergovernmental Panel on Climate Change [Core Writing Team, H. Lee and J. Romero (eds.)]. IPCC, Geneva, Switzerland. Tech. Rep., Intergovernmental Panel on Climate Change (IPCC) (2023). URL https://www.ipcc.ch/report/ar6/syr/. Edition: First.

[5] Stöcker, P. *et al.* Nuclear fission, natural gas, geothermal energy, nuclear fusion. the future role of baseload power plants (2025). URL https://en.acatech.de/publication/baseload-power-plants/download-pdf?lang=en. Series on “Energy Systems of the Futur” (ESYS).

[6] Kachirayil, F., Huckebrink, D., Bertsch, V. & McKenna, R. Trade-offs between system cost and supply security in municipal energy system design: An analysis considering spatio-temporal disparities in the Value of Lost Load. *Applied Energy* **381**, 124896 (2025). URL https://linkinghub.elsevier.com/retrieve/pii/S0306261924022797.
