# Supplementary material for: Author Correction: Long-duration electricity storage needs for coping with Dunkelflaute events in Europe
Source: Nat Commun. 2026 Jul 9;17:6035. doi: 10.1038/s41467-026-75342-9 (PMC13350742; doi:10.1038/s41467-026-75342-9)
Supplement: Supplementary file 1 — Original Fig. 2 [file 41467_2026_75342_MOESM1_ESM.pdf]

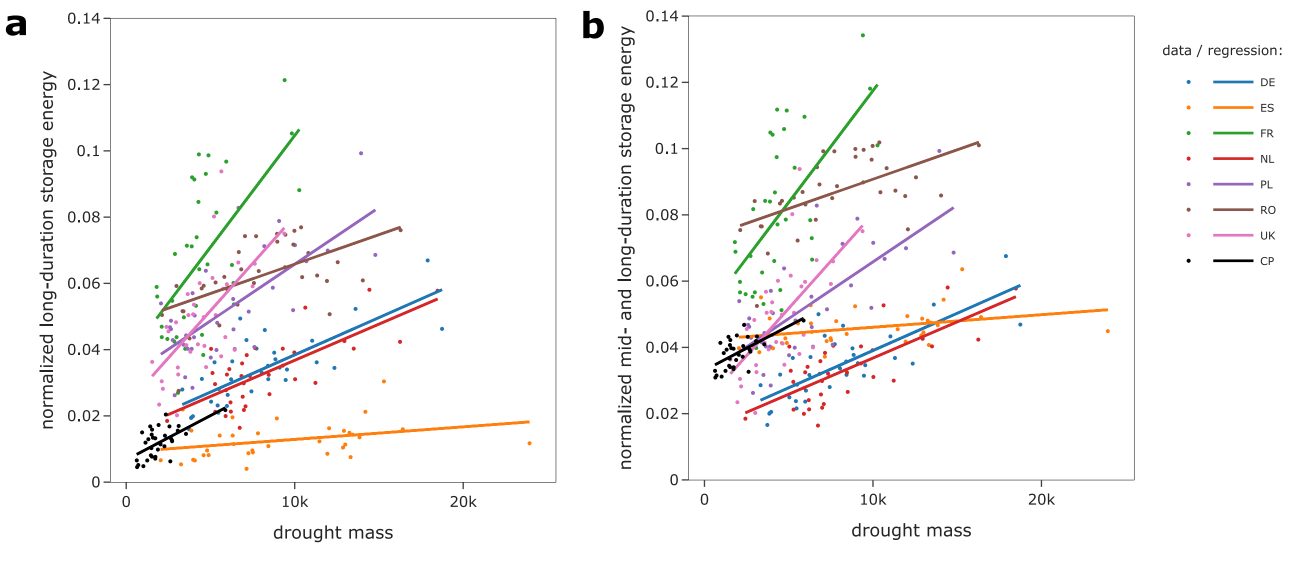

Fig. 2 | Correlation of the severity of most extreme winter drought events (drought mass) and normalized storage energy capacity. For comparison, we normalize the least-cost storage energy with the annual demand for electricity (including electrified heating) and hydrogen. For illustration, we exclude countries with least-cost storage energy below 5 TWh and countries with binding storage expansion potential constraints. Supplementary Fig. 3 shows the unfiltered regression results. We further include the pan-European copperplate scenario (CP). **a** Long-duration storage only. **b** Mid- and long-duration storage.
